# Styrene–Acrylic Emulsion with “Transition Layer” for Damping Coating: Synthesis and Characterization

**DOI:** 10.3390/polym13091406

**Published:** 2021-04-27

**Authors:** Daoyuan Chen, Mingjin Ding, Zhixiong Huang, Yanbing Wang

**Affiliations:** Key Lab of Advanced Technology for Specially Functional Materials, Ministry of Education, and School of Materials Science and Engineering, Wuhan University of Technology, Wuhan 430070, China; chendy@whut.edu.cn (D.C.); dingmj@whut.edu.cn (M.D.); zhixiongh@whut.edu.cn (Z.H.)

**Keywords:** styrene–acrylic, damping, core/shell, transition layer

## Abstract

In order to study the dynamic mechanical properties of styrene–acrylic latex with a core/shell structure, a variety of latexes were synthesized by semi-continuous seeded emulsion polymerization based on “particle design” with the same material. The latexes were characterized by rotary viscosimeter, dynamic light scattering (DLS), Fourier transform infrared spectroscopy (FTIR), transmission electron microscope (TEM), dynamic mechanical analysis (DMA), and universal testing machine. The effects of difference at the glass transition temperature (*T_g_*) of core and shell and the introduction of the “transition layer” on the damping and mechanical properties of latex film were studied. The results indicate that as the *T_g_* of core and shell gets closer, the better the compatibility of core and shell, from phase separation to phase continuity. Furthermore, the introduction of the “transition layer” can effectively improve the tensile strength and tan δ (max) of the latex film. The tensile strength and maximum loss factor (f = 1 Hz) of latex with the “transition layer” increased by 36.73% and 29.11% respectively compared with the latex without the “transition layer”. This work provides a reference for the design of emulsion for damping coating.

## 1. Introduction

Damping coatings are used for attenuating sound and damping the vibrations. The matrix resin is well known as the key factor for damping coatings to perform their damping function [1,2,3]. Generally, polymer chains can be in full motion near the glass transition temperature (*T_g_*), but the deformation cannot keep up with the change in the alternative stress [4]. Thus, internal consumption is severe, and the damping effect is strong. As a measure of material damping performance, the loss factor (tan δ) is the embodiment of material internal friction. Typically, when tan δ > 0.3, the material can achieve an effective damping performance [5,6].

Compared to the traditional solvent-based coatings, which contain a high quantity of organic solvents, waterborne coatings are becoming a popular alternative due to being free of VOC (Volatile Organic Compounds) [7,8]. Styrene–acrylic emulsion is a sort of polymer emulsion that introduces styrene into acrylate polymers. As a kind of coating matrix resin, the styrene–acrylic emulsion has outstanding advantages compared with ordinary acrylate emulsion products such as good adhesion, good water resistance, good heat resistance, and good aging resistance [9]. In comparison to acrylic emulsion, the styrene–acrylic emulsion has the characteristics of good performance and low price simultaneously. It is extensively used in adhesives [10], paper sizing machines [11], coatings [12,13,14,15], and other industries [16,17] due to its applicability and cost performance.

Core–shell emulsion polymerization originated with Okubo’s “particle design” theory, which was introduced in the 1980s [18]. By designing and modifying the particle structure, the emulsion properties were optimized with in the overall framework of the unchanged monomer composition. Several literatures and patents have studied the dynamic–mechanical and damping properties of core/shell latex particles [19,20,21]. Table 1 displays the reported literature results. In recent years, Zhiguo Li and his team have reported a series of methods to prepare controlled core–shell latex particles [22,23,24,25]. They explored the effects of monomer polymerization time, core–shell ratio, and amount of the transition layer on the morphological of the latex particles, and they focused on the morphology characteristics of the latex particles prepared using the transition layer mediated method. However, the damping properties of emulsions prepared in this method have not been studied. In addition, little has been done concerning the damping properties with the same total amount of material.

In this work, in order to investigate the link of *T_g_* of core/shell about dynamic–mechanical and especially damping properties, four latex particles (CS1, CS2, CS3, CS4) with the same formula were synthesized by pre-emulsifying semi-continuous seeded emulsion polymerization out of “particle design”. Furthermore, in combination with the damping mechanism of polymer material, the “transition layer” poly (butyl methacrylate) (PMBA) was introduced into four latex particles (CST1, CST2, CST3, and CST4) by using the same formula. The samples were characterized by rotary viscosity meters, dynamic light scattering (DLS), Fourier transform infrared spectroscopy (FTIR), transmission electron microscope (TEM), dynamic thermal mechanical analysis (DMA), and the universal testing machine. The effects of the difference at *T_g_* between the core and shell and the introduction of the “transition layer” on the damping and mechanical properties of latex film were compared and analyzed. It is worth saying that the Fox equation was used in the formulation of *T_g_* of core/shell as the theoretically *T_g_*. Equation (1) is as follows [20].

(1) $$1/T_{g}=w_{1}/T_{g1}+w_{2}/T_{g2}$$

where *w*_1_ and *w*_2_ are weight fractions of components 1 and 2, respectively.

## 2. Materials and Methods

### 2.1. Materials

Styrene (St), methyl methacrylate (MMA), n-butyl acrylate (BA), butyl methacrylate (MBA), initiator potassium persulfate (KPS), and sodium bicarbonate (NaHCO_3_) were purchased from Sinopharm Group Chemical Reagent Co. LTD (Shanghai, China) at reagent grade. Emulsifiers, sodium dodecyl benzene sulfonate (SDS) and alkylphenol polyoxyethylene ether (OP-10), were available from Aladdin (Shanghai, China) and Tianjin Photonics Reagent Co. LTD (Tianjin, China), respectively. Deionized water (DDI) was used in all experiments. All the chemicals were used directly without further purification.

### 2.2. Latex Preparation

#### 2.2.1. Synthesis of Seed Latex

Deionized water, emulsifiers (SDS, OP-10), and pH buffer (NaHCO_3_) were added in a 500 mL four-neck flask equipped with a mechanical stirrer, constant pressure titration funnel, thermometer, and condensate tube. The flask was placed in the water bath with a temperature of 65 °C and stirred at 350 rpm for 20 min at least. Then, the mixed monomers were added into the flask for pre-emulsification for about 30 min; after that, the temperature was raised to 75 °C, while the rotating speed was adjusted to 250 rpm, and 20 g KPS aqueous solution was added. Thus, the seed emulsion with blue light was obtained after 30 min.

#### 2.2.2. Synthesis of Core–Shell Latex

The mixed monomer was added dropwise into the seed emulsion at a constant rate (0.47 g/min) using the constant pressure titration funnel. Then, 10 g of KPS aqueous solution was added dropwise (2 g/min) after 30 min. After the monomer was added, the core emulsion was obtained for another 1 h at 80 °C reactions.

After that, part of the “transition layer” monomer was added with a relatively rapid rate (0.6 g/min) within 20 min, and it was kept for 10 min after the addition was completed. Then, the remaining shell monomer was added to the constant pressure titration funnel to drip with a slower rate (0.5 g/min) within 130 min, while adding 10 g of KPS aqueous solution.

**Scheme 1:**
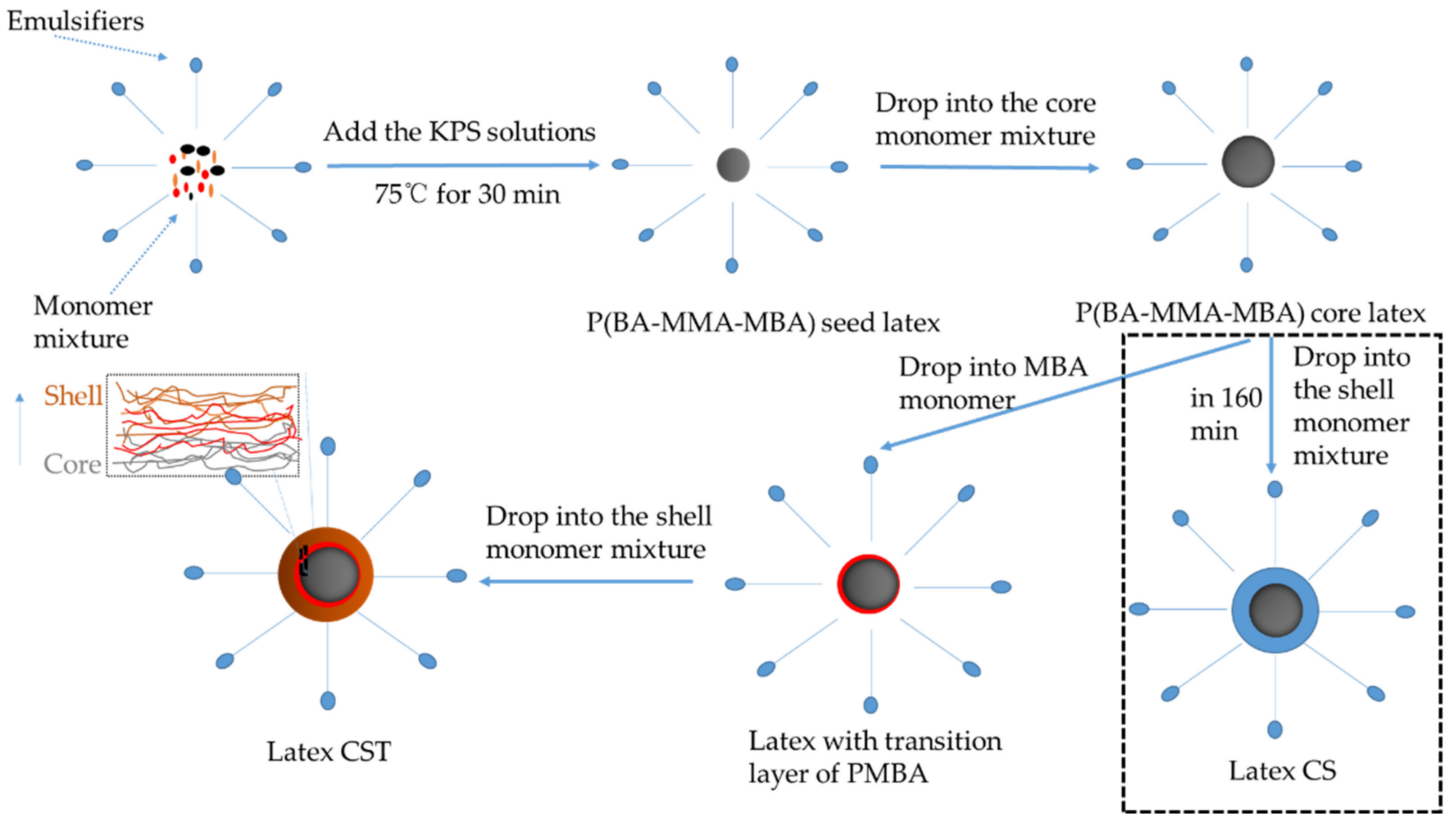
Synthesis process of experimental design.

During the reaction, a certain amount of the KPS aqueous solution was added every 30 min until depletion. Once the dripping monomer was completed, the temperature was raised to 85 °C and kept for 1 h [22,23,24,25]. The bottle was taken out when the temperature decreased to about 40 °C, and the 100 mesh gauze was filtered to obtain the C/S emulsion for later use. Recipes for the synthetic emulsion in this study are listed in Table 2. The fabrication process of experimental design for the latex particle of CST is shown in Scheme 1.

### 2.3. Characterization

The emulsions were poured into a clean polytetrafluoroethylene groove mold, and its thickness was controlled. The emulsion was placed in a drying oven at 25 °C for 7 days. As the water evaporated, the latex particles stayed close together, and the film was formed.

The solid content, gel rate, and conversion of the obtained emulsions were measured via the gravimetrical method. The viscosity of the emulsion was measured with a rotational viscometer (NDJ-8S, Shanghai, China).

The damping property was obtained by dynamic thermomechanical analysis (DMA/SDTA 861e METTLER TOLEDO, Switzerland). The samples were made into a disc with a diameter of 10 mm and a thickness of about 2 mm with a puncher. The shear mode was used over a temperature range from −25 to 125 °C at a heating rate of 3 °C/min; multi-frequency mode was selected for test frequency (1, 10, 25, 50, 100 Hz).

The universal testing machine (Instron 5967, Instron, Norwood, MA, USA) was used to measure the tensile properties. Dumbbell-like samples were prepared with a gauge length of 33.00 mm and a width of 6.00 mm according to GB/T 1040–2006, with a tensile rate of 100 mm/min. Make sure that the long axis of the sample and the axis of the testing machine are in a straight line.

Scanning electron microscope (SEM) analysis was carried out on a JEM-S4800 SEM (JEOL, Tokyo, Japan). The cross-sectional images of the tensile test specimens were obtained by SEM.

The latex particles were diluted with deionized water to adjust the solid content about 0.01 wt % and then measured by a dynamic light scattering (DLS) (Mastersizer 2000/Mastersizer 2000, Malvern, UK).

## 3. Results and Discussion

### 3.1. The Basic Properties of the Emulsion

Table 3 provides a detailed summary of the characteristics of the synthesized emulsions. The particle size and viscosity of the emulsions are about the same. This may be due to the fact that the experimental methods, reaction time, and the total amount of materials in the emulsions are completely the same. The solid content of produced emulsions is 44.11–44.73%, which is basically consistent with the theoretical solid content of 45.40%. Thus, the conversion of the monomer is about 98% and is combined with the FTIR spectra, which indicate that during the synthesis of the emulsion, the monomers have substantially participated in the polymerization reaction and the reaction is completed. Due to the stage heating, the semi-continuous feeding and the intermittent addition of initiator polymerization methods were used to ensure that the emulsion polymerization rates during the reaction could be consistent. As expected, the emulsion obtained by the reaction has almost no gel product.

**Figure 1:**
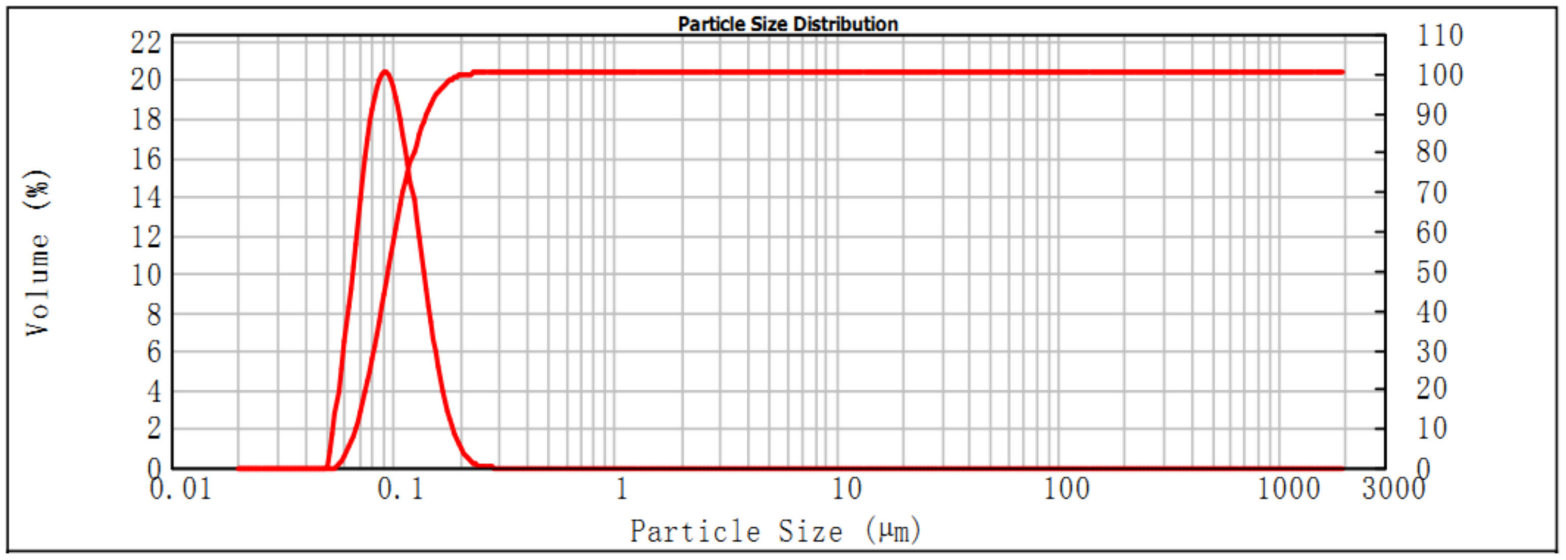
The representative DLS of latex.

**Figure 2:**
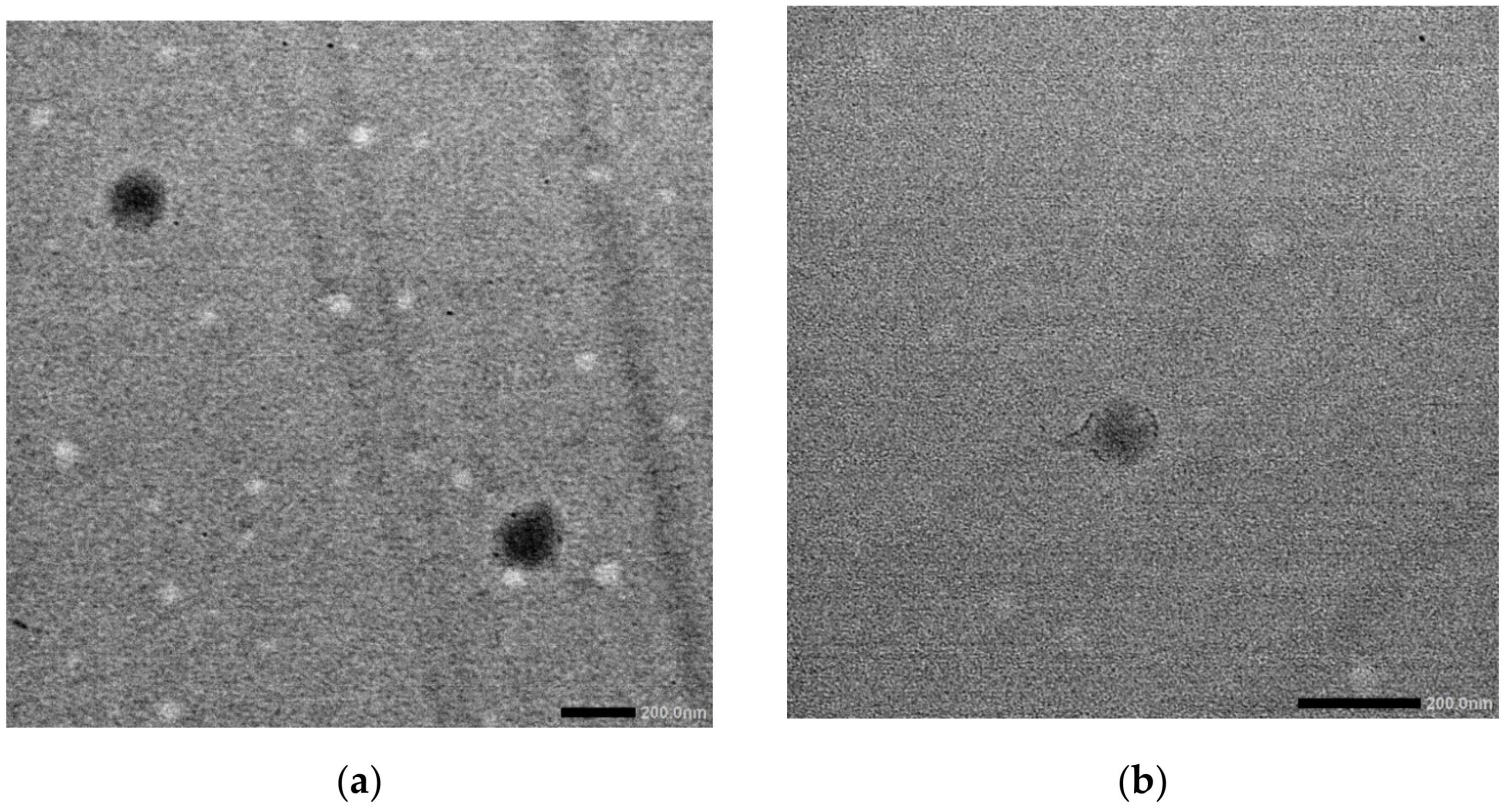
TEM images of latex particles (**a**) CS4, (**b**) CST4.

Combining with DLS (Figure 1) and TEM, the particle size of the latex particles is about 100 nm. Figure 2 shows the TEM images of latex CS4 and CST4. It can be seen that the morphology of the latex particles was sphere-like, the darker inner and the light-colored external might be because of the core–shell structure.

### 3.2. Damping Properties of the Latex Film

In this study, the damping properties of the samples were measured by DMA.

**Figure 3:**
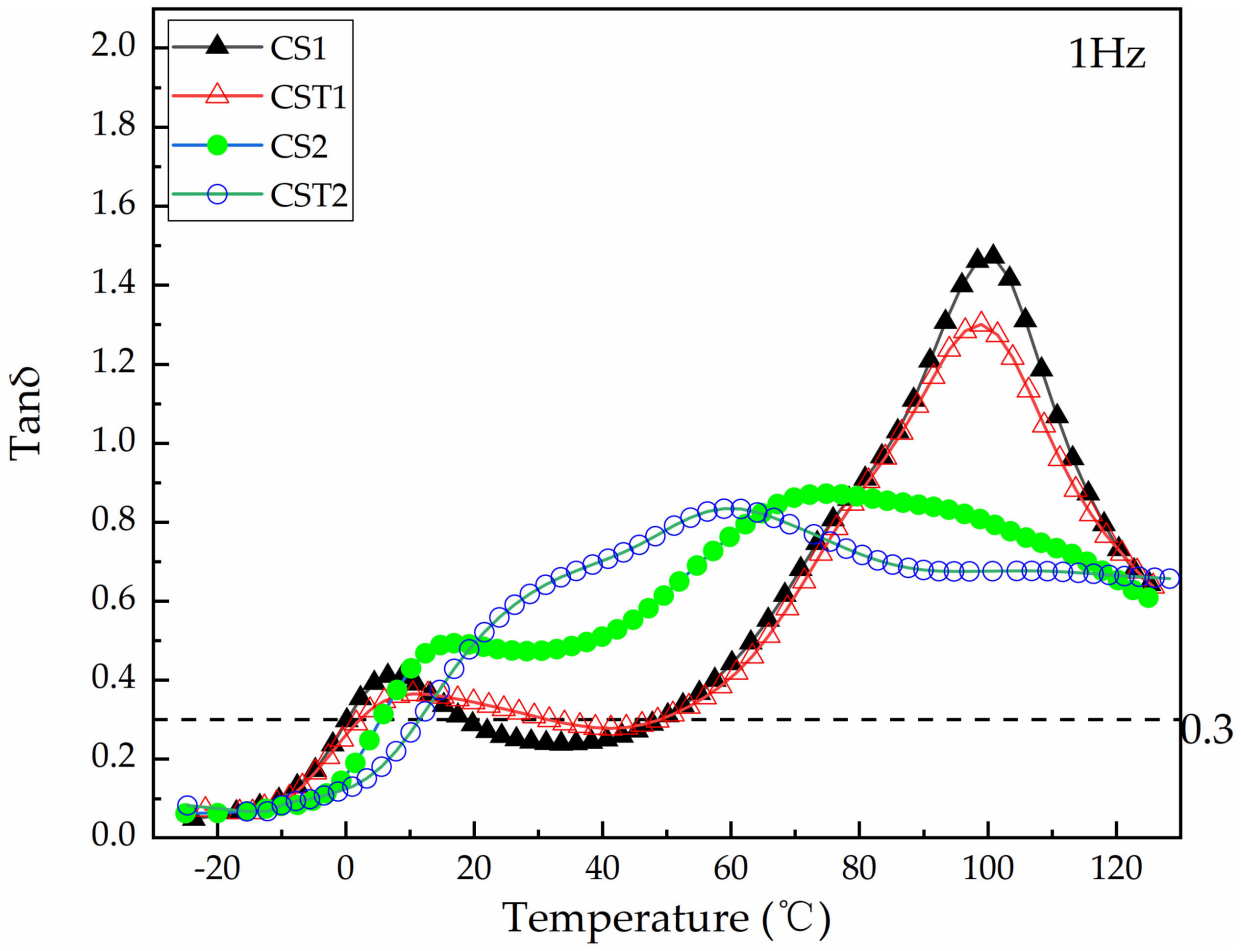
The loss tangent versus temperature curves of CS1, CS2, CST1, and CST2 at 1 Hz.

**Figure 4:**
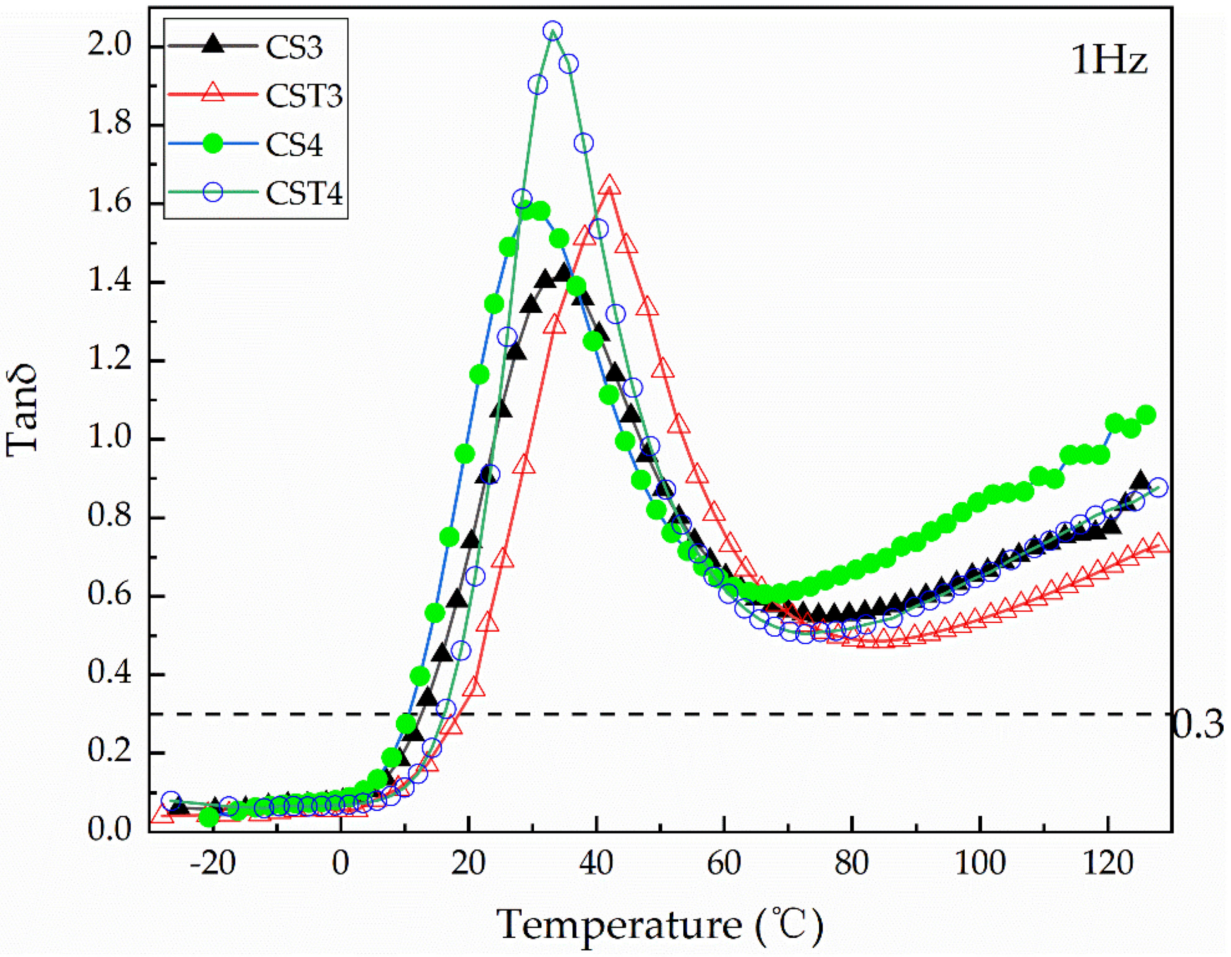
The loss tangent versus temperature curves of CS3, CS4, CST3, and CST4 at 1 Hz.

The temperature–tan δ curves of CS1, CS2, CST1, and CST2 at 1 Hz are shown in Figure 3. The temperature–tan δ curves of CS3, CS4, CST3, and CST4 at 1 Hz are shown in Figure 4. Table 4 presents the *T_g_* of each layer for all samples based on the Fox equation and the damping parameters of samples. In the following, *T_g_* stands for the temperature of the loss peaks. The theoretical *T_g_* was calculated by FOX.

As can be seen from Figure 3, two distinct characteristic peaks appear on the CS1 curve, in which 100.84 °C is attributed to the core polymer of the latex particle, and 6.55 °C is attributed to the shell polymer. The theoretical *T_g_* of the core and the shell polymer of CS1 are 96.07 °C and −13.31 °C, respectively. Similarly, CS2 also shows a similar curve to CS1, while both peaks (74.83 °C and 16.84 °C) are closer to each other, and the curve between both peaks show a platform structure. On the curves of CST1 and CST2, the same phenomenon occurs.

It is known by reference [29] that the *T_g_* obtained from the temperature–tan δ curve is higher than that of other test methods such as DSC, TMA, etc. The tan δ peak of the high-temperature part has shifted to the left, and the low-temperature part has shifted to the right, and the two peaks are distinct. Obviously, the phase separation happened in CS1 and CST1.

In Figure 3, it can be found that the two tan δ peak values of CST1 that contain the “transition layer” have decreased slightly, whereas the corresponding *T_g_* remains almost unchanged. This may be due to the fact that the composition of the core polymer is the same, and also the *T_g_* of the core part is higher than the reaction temperature, and it is in a glassy transition state during the subsequent reaction. It also can be found that the *T_g_* at the low temperature of CST1 shifted to the right, the tan δ peak value decreased, and the transition between both peaks became smoother. Two effects may have caused the phenomenon. Firstly, by comparing with CS1, the addition of the “transition layer” MBA of CST1 resulted in the *T_g_* reduction of the shell layer. Secondly, after blending the shell of CST1 with the “transition layer”, PMBA causes the *T_g_* to shift to the right. A similar phenomenon can be seen in the comparison of CS2 and CST2; only the changes between the two peaks of the CS2 and CST2 curves are smoother. It is worth noting that CST2 combines the core layer and the shell layer due to the “bridge” functions as the “transition layer”, which can be interpreted as forming a latex interpenetrating polymer network (LIPN) structure [6,20,21].

From Figure 4, it can be observed that as the *T_g_* of the core polymer decreases, the total number of materials remains unchanged. In other words, the *T_g_* of the shell part also increases accordingly, and the difference at *T_g_* between the core and the shell is narrowing; thus, only one peak is observed on the curve. This indicates that unlike CS1 and CS2, phase separation has not occurred in CS3 and CS4, and better compatibilities appear in the core and shell. The same applies to the curves of CST3 and CST4.

The temperature–tan δ curves of CS3, CST3, CS4, and CST4 are all single peaks, which indicates that the shell polymer is more easily compatible with the core polymer without phase separation. By further comparison, the *T_g_* of CST3 is higher than that of CS3, and such cases also occurred in CST4 and CS4. It might be explained that compared with the core–shell structure without the “transition layer”, the “transition layer” is a homopolymer composed of a single monomer with moderate *T_g_*, and the solubility parameter is closer to the core and shell polymers. Therefore, physical entanglement is more likely to occur in macromolecules, making the intramolecular motion more obstructed, and then it shifts the tan δ peak to the right.

There are two possible reasons for the single peak. Firstly, as the difference at *T_g_* between the core and shell gets smaller (from 109.38 °C of CS1 to 16.04 °C of CS4), the compatibility between the core and shell improved. In addition, it may also be because the reaction temperature is 75–85 °C. During the reaction process, CS3 and CS4 were in a viscous flow state, the monomer and the core were more compatible, and the boundary between the two phases becomes more blurred due to the phase continuity formed, which shows a single peak on the temperature–tan δ curve.

Reflecting on the damping performance, due to more obstacles in molecular motion, there is both an increase in the physical cross-linking network and internal friction between molecules. Compared with CS3 and CS4, the tan δ_max_ of CST3 and CST4 respectively increased by 15.49% and 29.11%. However, the effective damping temperature (tan δ > 0.3) range did not significantly improve compared to CS4 and CST4; this might be because the chain of the “transition layer” does not significantly affect the internal friction in low and high temperature (glassy state and viscous state).

### 3.3. Activation Ennergy of Latex in Glass Trasition

**Figure 5:**
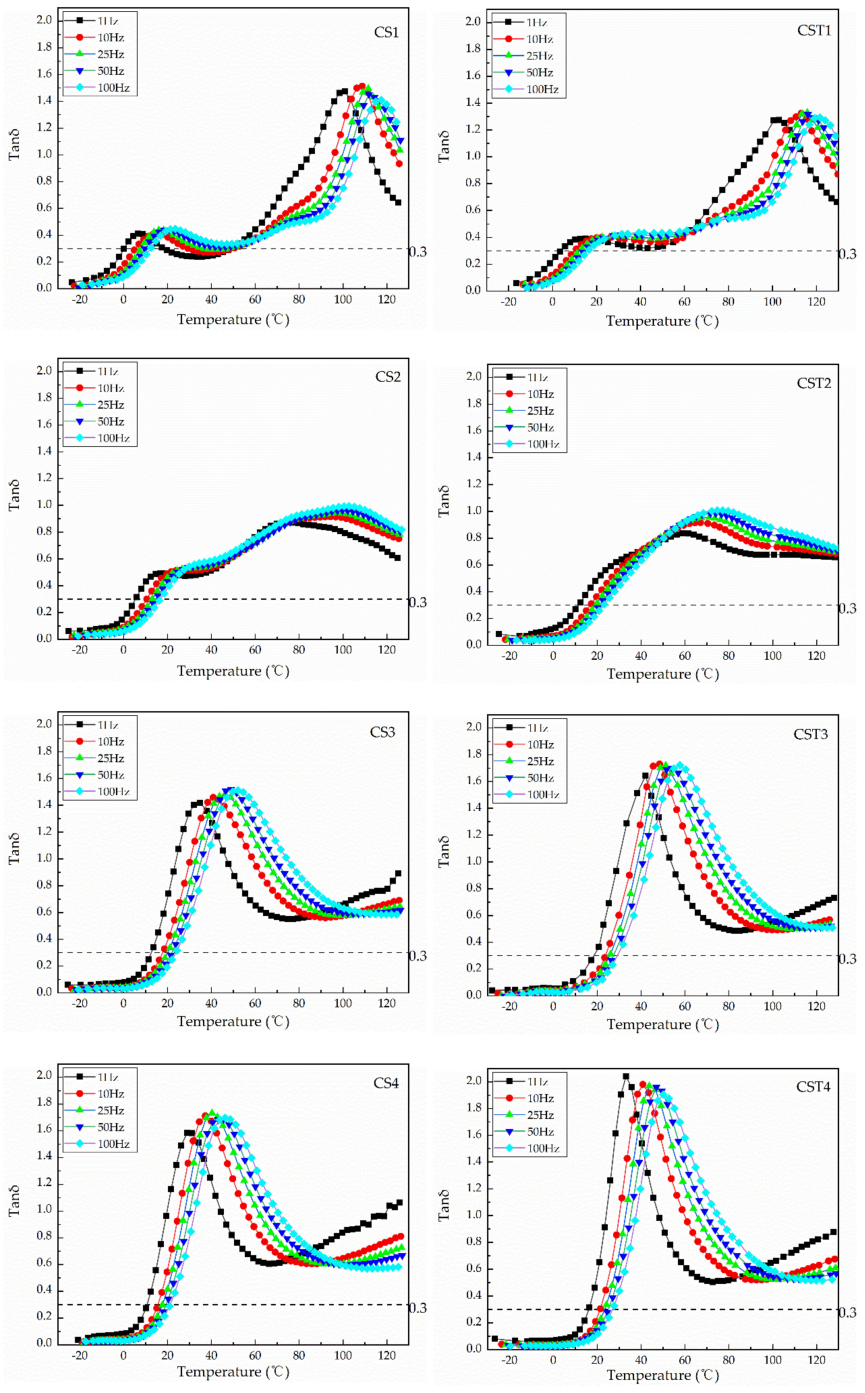
The loss tangent versus temperature curves of latex about multi-frequency.

Figure 5 shows the temperature–tan δ curves of latexes in 1, 10, 25, 50, and 100 Hz. It is well known that the dynamic mechanical behavior of viscoelastic damping materials is directly related to time (deformation frequency) and temperature. It can easily be seen that when the frequency increases from Figure 5, the curve moves toward the high-temperature direction. This is consistent with Time–Temperature Superposition (TTS).

According to the relationship between the experimental frequency ω and the obtained transition temperature *T* (absolute temperature, K),

(2) $$\omega =\omega_{0}e^{-\Delta E/RT}.$$

In the formula, Δ*E* is the activation energy of the corresponding moving unit, J/mol, *R* = 8.314 J/mol/°C, which is a constant. ω is the measurement frequency, Hz. Take the logarithm of both sides of the above formula to get [30,31]:

(3) $$\ln\omega =\ln\omega_{0}-\frac{\Delta E}{RT_{g}}.$$

**Figure 6:**
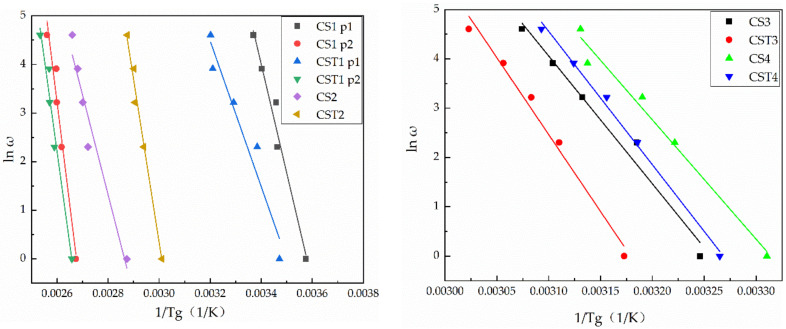
Plot of ln (ω) versus (1/*T_g_*) of latex.

By making the curve (linear fitting) of 1/*T_g_*–ln ω, the slope of the curve obtained is—Δ*E*/*R*, which is shown in Figure 6. Table 5 shows the corresponding *T_g_* of latex particles at different frequencies (the corresponding temperature of the peaks). Figure 6 shows the curve of 1/*T_g_*–ln ω. By comparing the activation energy of CS1, CS2, CS3, and CS4, CST1, CST2, CST3, and CST4, it was found that as the *T_g_* between the core and shell becomes closer, the activation energy required for the latex particles to undergo glass transition increases. This is consistent with the aforementioned *T_g_* of CS4 being higher than that of CS3.

By comparing the series of CS and CST, it can be found that the *T_g_* activation energy of CS1 and CS2 is higher than that of CST1 and CST2, respectively. This means that it is harder for CS1 and CS2 to transition to the rubbery state. It could be explained by the introduction of the “transition layer” making the chains of polymers easier to move and reducing the difference at *T_g_* between the core and shell. On the contrary, the *T_g_* activation energy of CST3 and CST4 is higher than that of CS3 and CS4, respectively. This indicates that the chains of CST3 and CST4 need more energy to move in the glass transition, and this is consistent with the aforementioned *T_g_* of CST4 being higher than that of CS4, which has been explained in Section 3.2.

### 3.4. Tensile Strength of Latex Film

**Figure 7:**
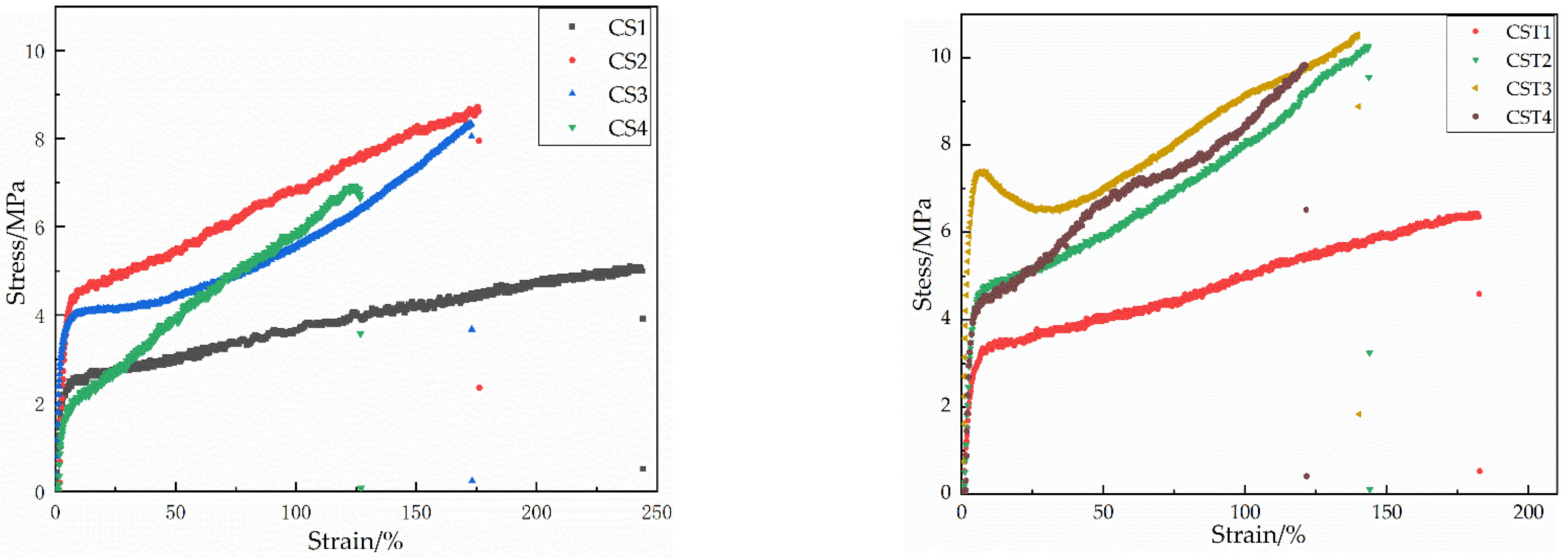
Stress–strain curves of the latex film (one of samples each latex).

The stress–strain curves of the latex film are shown in Figure 7, and Table 6 shows the tensile strength and elongation at break of latex film. It is found that with the decrease of the *T_g_* difference at *T_g_* between the core and shell, the tensile properties of the latex films increase first and then decrease, but the elongation at break keeps decreasing. The introduction of the “transition layer” does not change this trend.

There are two possible reasons for this. On the one hand, as the core *T_g_* decreases, the amount of St in the shell polymer increases, and the amount of soft monomer decreases, which results in the hardening of the shell polymer. At this level, the tensile strength of the polymer is improved. On the other hand, as the *T_g_* of the core polymer decreases, the core polymer becomes soft, and the soft core has a weakening effect on the mechanical properties of the latex film. Under the combined action of the above two, the tensile strength of the latex film increases firstly and then decreases. In addition, it could be obtained a latex film with the best mechanical properties by adjusting the *T_g_* difference between the core and shell.

From Figure 7, it can also be found that the tensile strength of the latex film with the “transition layer” structure is improved to different degrees than the latex film without the “transition layer” structure, 20.53%, 17.09%, 24.61%, and 36.73% respectively. This indicates that the introduction of the “transition layer” can effectively improve the tensile strength of the latex film under the same formulation. This may be because the introduction of the “transition layer” makes the connection between the core and the shell closer, and the interface between the core and the shell is stronger. This can also be confirmed from the comparison of the activation energy in Table 5. Compared with the CS series without a “transition layer”, the elongation at break of the CST series latex films is reduced. That can be interpreted as the introduction of the “transition layer” having a positive effect on the compatibility between the core and shell of the latex films. In addition, compared with CS, the proportion of ST in the outermost layer of CST is higher, which limits the inter–chain motion of some molecules, leading to the decrease of elongation at break.

**Figure 8:**
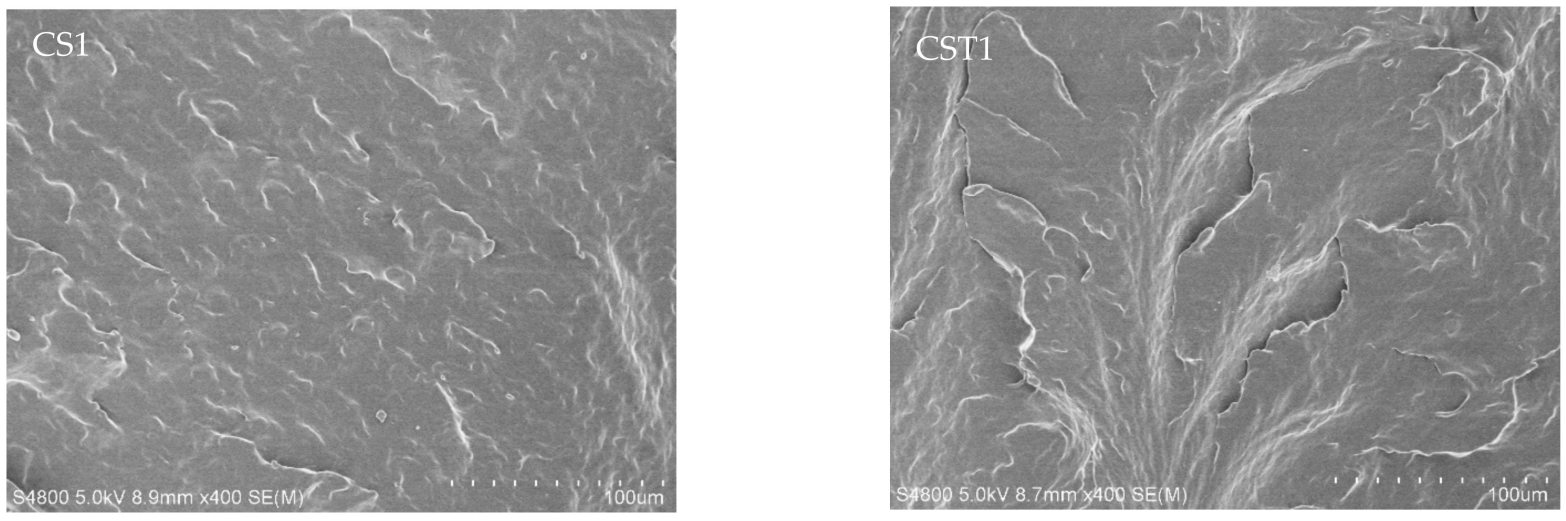

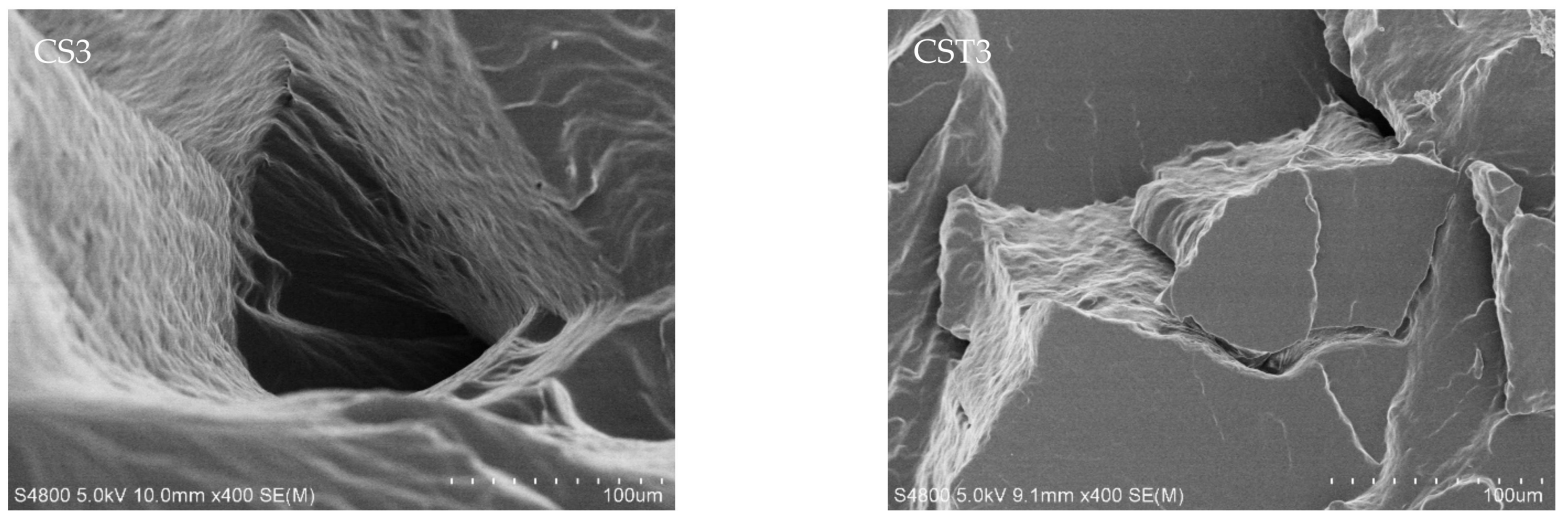
The cross-sectional SEM images of the tensile test specimens.

CST3 exhibits an obvious yielding process, and CS3 also exhibits a yielding process from the stress–strain curves in Figure 7. To further analyze the reasons for the significant difference in the shape of tensile stress–strain curves, SEM was used to observe the cross-sectional images of the tensile test specimens. As shown in Figure 8, CST3 and CS3 exhibit a much more rough with shear deformation. Shear bands with higher roughness lead to longer crack propagation paths, which prevents deformation and crack growth and results in higher mechanical strength values. Compare CS1 and CST1 as well as CS3 and CST3. For the cross-sectional images of the tensile test specimen latex film, the “transition layer” also exhibits a relatively rough surface, which results in higher mechanical strength values.

## 4. Conclusions

In this work, a variety of different core–shell emulsions were prepared by adjusting the difference at *T_g_* between the core and shell using the same formula. It was found that the basic properties of the emulsion such as conversion, viscosity, and particle size are not much different. Mechanical tests show that as the difference at *T_g_* between the core and shell decreases, the tensile properties of the latex film increase first and then decrease. The DMA results show that as the difference at *T_g_* of the core and shell polymer decreases, the compatibility of core and shell improved, from phase separation to phase continuity. It can effectively improve the tensile strength and tan δ max of the latex film through the introduction of the “transition layer”. The tensile strength and maximum loss factor (*f* = 1 Hz) of latex particles CST4 are increased by 36.73% and 29.11% respectively compared with CS4, but the damping temperature range does not change much.

Therefore, this work may offer some possibilities for preparing some different core–shell emulsions by adjusting the *T_g_* of the core–shell structure to obtain different properties under the same formula.

## Figures and Tables

**Table 1:** The results of the reported.

Sample	Material	tan δ_max_	Effective Temperature Region (tan δ > 0.3) (°C)	*T_g_*	Tensile Strength/MPa	Elongation at Break/%
Literature1 [20]	styrene–acrylic emulsion	0.88	−12.0 to 97.1	≈6	-	-
Literature2 [21]	styrene–acrylic emulsion	0.83	−23.7 to 124	-	-	-
Literature3 [26]	polyurethane/polyacrylate with graphite particles	0.528	−1 to 108	55	0.540	182
Literature4 [27]	polyurethane–acrylate emulsions:	≈0.7	−26.6 to 79.14	≈−8	≈1.6	≈215
Literature5 [8]	polyurethanes–acrylate	1.23	−11 to 64	10.5	-	-
Literature6 [28]	polyurethane–acrylate	0.66	−15 to 68	≈4	-	-

**Table 2:** The recipes used for the synthesis of core/shell latexes.

Sample		CS1	CS2	CS3	CS4	CST1	CST2	CST3	CST4
Seed	Water (g)	60	60	60	60	60	60	60	60
	SDS (g)	2.00	2.00	2.00	2.00	2.00	2.00	2.00	2.00
	OP-10 (g)	1.00	1.00	1.00	1.00	1.00	1.00	1.00	1.00
	NaHCO3 (g)	0.30	0.30	0.30	0.30	0.30	0.30	0.30	0.30
	BA (g)	0	0	0.75	2.25	0	0	0.75	2.25
	MMA (g)	3.25	3.25	3.25	3.25	3.25	3.25	3.25	3.25
	MBA (g)	0.75	2.25	2.25	0.75	0.75	2.25	2.25	0.75
	St (g)	2.25	0.75	0	0	2.25	0.75	0	0
	KPS (solutions/g) ^b^	20	20	20	20	20	20	20	20
Core	BA (g)	0	0	2.25	6.75	0	0	2.25	6.75
	MMA (g)	9.75	9.75	9.75	9.75	9.75	9.75	9.75	9.75
	MBA (g)	2.25	6.75	6.75	2.25	2.25	6.75	6.75	2.25
	St (g)	6.75	2.25	0	0	6.75	2.25	0	0
	KPS (solutions/g) ^b^	10	10	10	10	10	10	10	10
C/S ^a^	MBA (g)	22	16	16	22	*12*	*12*	*12*	*12*
						*10*	*4*	*4*	*10*
	BA (g)	38	38	35	29	38	38	35	29
	St (g)	15	21	24	24	15	21	24	24
	KPS (solutions/g) ^b^	35	35	35	35	35	35	35	35

^a^: In the core–shell stage, all the mixed monomers are added to CS series at one time, and 12 g MBA was added to latex of CST series separately at first, followed by the remaining mixed monomers. ^b^: every 10 g KPS solutions contains 0.1 g KPS.

**Table 3:** Characteristics of the core/shell latex.

Samples	Solid Content/%	Conversion/%	Viscosity/mPa·s	Particle Size ^a^/nm
CS1	44.73	98.51	203	~100
CS2	44.28	97.54	201	
CS3	44.52	98.06	201	
CS4	44.11	97.16	199	
CST1	44.61	98.33	205	
CST2	44.46	97.94	203	
CST3	44.44	97.88	198	
CST4	44.70	98.46	202	

^a^: Obtained by DLS and TEM.

**Table 4:** The damping properties of synthesized latex particles.

**Sample with “Transition Layer”**	**Mass Ratio**	***T_g_* (Fox)/°C**	***T_g_* (tan δ_max_)/°C**	**Tan δ_max_**	**Effective Temperature Region (tan δ > 0.3) (°C)**
	**Core:Shell**	**Core/Shell**			
CS1	1:3	96.07/−13.31	100.84/6.55	1.47	0.31–18.63/49.37–~
CS2	1:3	69.18/−9.30	74.83/16.84	0.87	5.28–~
CS3	1:3	44.61/−3.95	34.92	1.42	12.75–76.99 ^a^
CS4	1:3	18.94/2.90	28.92	1.58	10.57–66.56
**Sample without “Transition Layer”**	**Core:Shell (Transition Layer:Shell)**	**Core/Transition Layer/SHELL**	***T_g_* (tan δ_max_)/°C**	**Tan δ_max_**	**Effective Temperature Region (tan δ > 0.3) (°C)**
CST1	1:3 (12:63)	96.07/20.00/−18.82	99.00/10.47	1.30	2.50–30.74/49.37–~
CST2	1:3 (12:63)	69.18/20.00/−14.23	61.91/23.93	0.83	11.51~
CST3	1:3 (12:63)	44.61/20.00/−8.07	42.04	1.64	18.63–85.24 ^a^
CST4	1:3 (12:63)	18.94/20.00/−0.14	33.13	2.04	15.85–73.86

^a^: The loss and temperature curve of some polymers will become uneven and rise in the high temperature region. It might be that these polymers may enter a viscous state in high temperature areas. Thus, before these polymers enter a viscous state, all effective temperature regions are quoted.

**Table 5:** *T_g_* and Δ*E* of latex.

Sample with “Transition Layer”	Frequency/Hz	*T_g_*/°C	Δ*E* (KJ/mol)	Sample without “Transition Layer”	Frequency/Hz	*T_g_*/°C	Δ*E* (KJ/mol)
CS1	1	6.55/100.84	186.31/351.89	CST1	1	14.92/103.06	124.57/307.94
	10	15.68/108.81			10	22.33/113.05	
	25	16.00/111.57			25	30.62/115.80	
	50	20.80/111.88			50	38.38/116.09	
	100	23.63/117.40			100	39.18/121.55	
CS2	1	34.92	317.91	CST2	1	58.91	278.66
	10	40.84			10	67.03	
	25	46.10			25	71.09	
	50	49.03			50	71.60	
	100	52.13			100	74.71	
CS3	1	34.92	215.33	CST3	1	42.04	259.24
	10	40.84			10	48.39	
	25	46.10			25	51.19	
	50	49.03			50	54.05	
	100	52.13			100	57.66	
CS4	1	28.92	200.9	CST4	1	33.13	224.78
	10	37.25			10	40.76	
	25	40.3			25	43.72	
	50	45.58			50	46.95	
	100	46.27			100	50.16	

**Table 6:** Tensile strength and elongation at break of latex film.

Sample		Tensile Strength/MPa	Elongation at Break/%	Sample		Tensile Strength/MPa	Elongation at Break/%
With “transition layer”	CS1	5.21	243.2	Without “transition layer”	CST1	6.28	182.1
	CS2	8.72	175.6		CST2	10.21	143.6
	CS3	8.28	172.3		CST3	10.32	139.8
	CS4	6.96	126.4		CST4	9.95	121.4

## Data Availability

The data presented in this study are available on request from the first author.
